# Neuralgic Rheumatism

**Published:** 1831

**Authors:** 


### NEURALGIC RHEUMATISM.

M. CONDRET relates a curious case in a late number of the JOURNAL COMPLEMENT, which we shall here briefly notice. A spanish colonel, an unfortunate exile from his native land, and forced to quit the land of liberty—France, took up his abode in England. But the climate of this country disagreed with his health. He became subject to violent rheumatic pains alternating with colic, diarrhœa, and much gastric derangement. By a singular fatality a number of bugs got into his right ear, and one of them remained and died in that asylum. From this moment the colonel was harrassed with pains round the base of the skull, but especially in the vicinity of the right ear, whence it seemed to radiate to other parts. The original rheumatic pains in his right arm and shoulder now ceased. His hair began to turn white and fall off, while a great number of small but dense tubercles formed on various parts of the scalp. Sir Astley Cooper and many other eminent surgeons were consulted, but without affording any relief. At length a quack was resorted to, who threw in an injection of soap and water into the meatus auditorius, and washed out a dead bug. The pains about the ear instantly ceased, and those in the other parts of the head were greatly diminished. Returning to France, the colonel consulted M. Condret for a troublesome itching at the bottom of the meatus auditorius. The scalp was still covered with tubercles, some of them ulcerated. On carefully examining the ear, M. C. thought he could discover something unnatural down on the membrana tympani. On introducing an instrument he extracted a small ball, composed of short hairs—hairs, in fact, which grew in the meatus, but which had been detached, from time to time, when the hair was falling from the scalp. The tubercles now rapidly disappeared—the itching of the meatus ceased—but now the original rheumatism of the right arm and shoulder resumed its post, and continued to annoy the unfortunate Spaniard. Finding no benefit from medicine, the colonel took it into his head one day, to apply a handkerchief, in the form of a tourniquet, tightly round the affected arm. The part below the ligature swelled and became somewhat œdematous; but from that time the rheumatism ceased. Thus, then, the lucky thought of a Charlatan, and the unpromising remedy resorted to by the patient himself, were more effectual than all the resources of science procured from the most skilful of the faculty!

---

